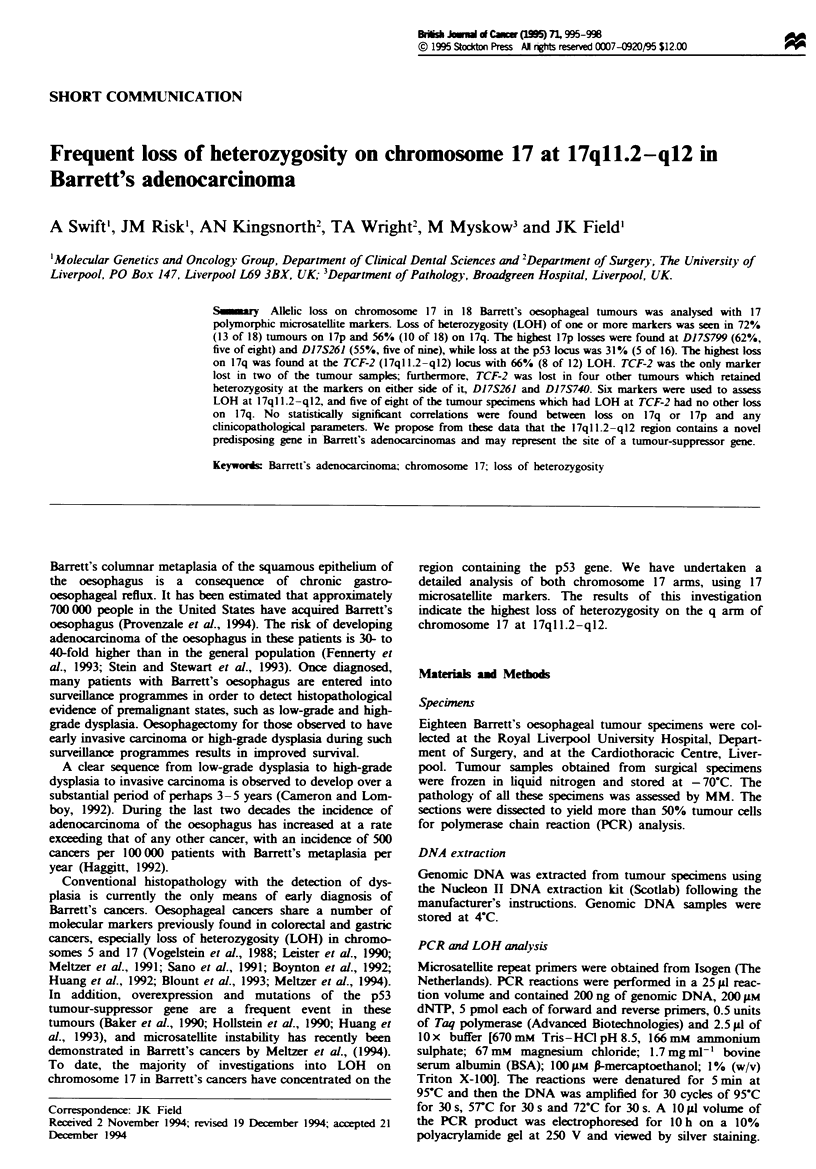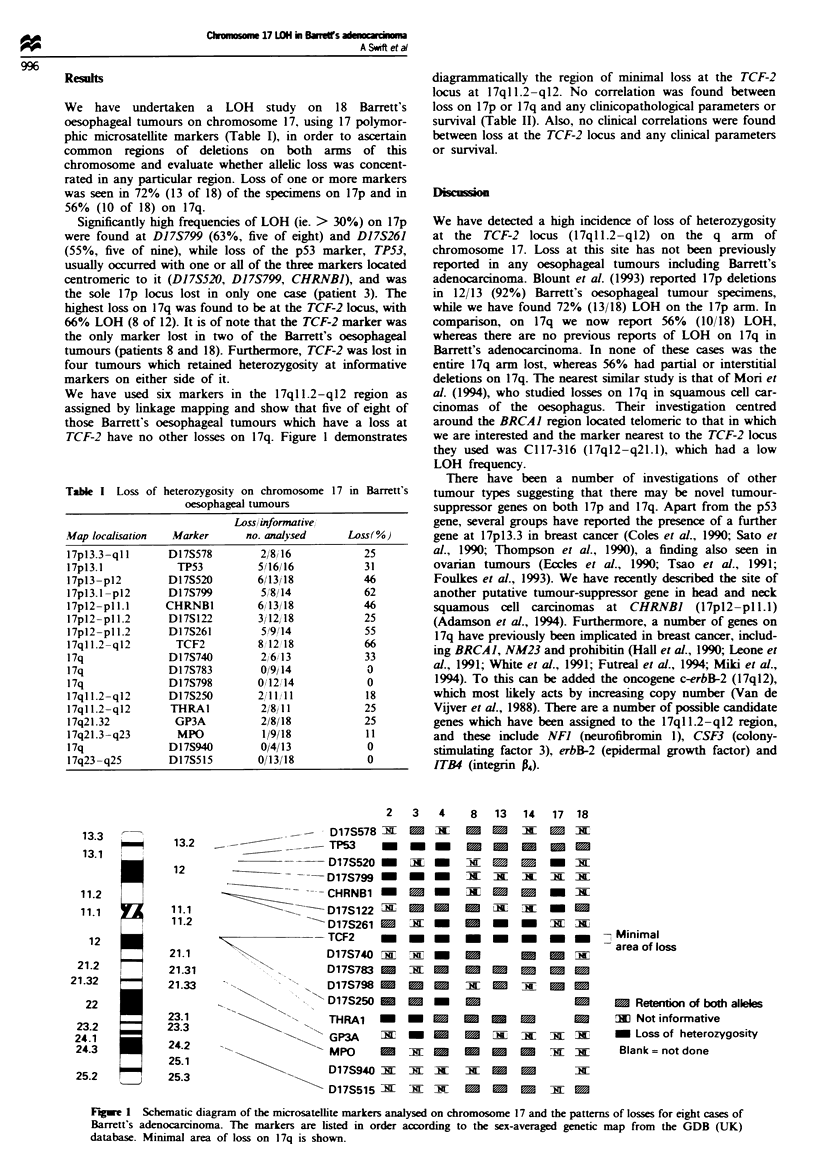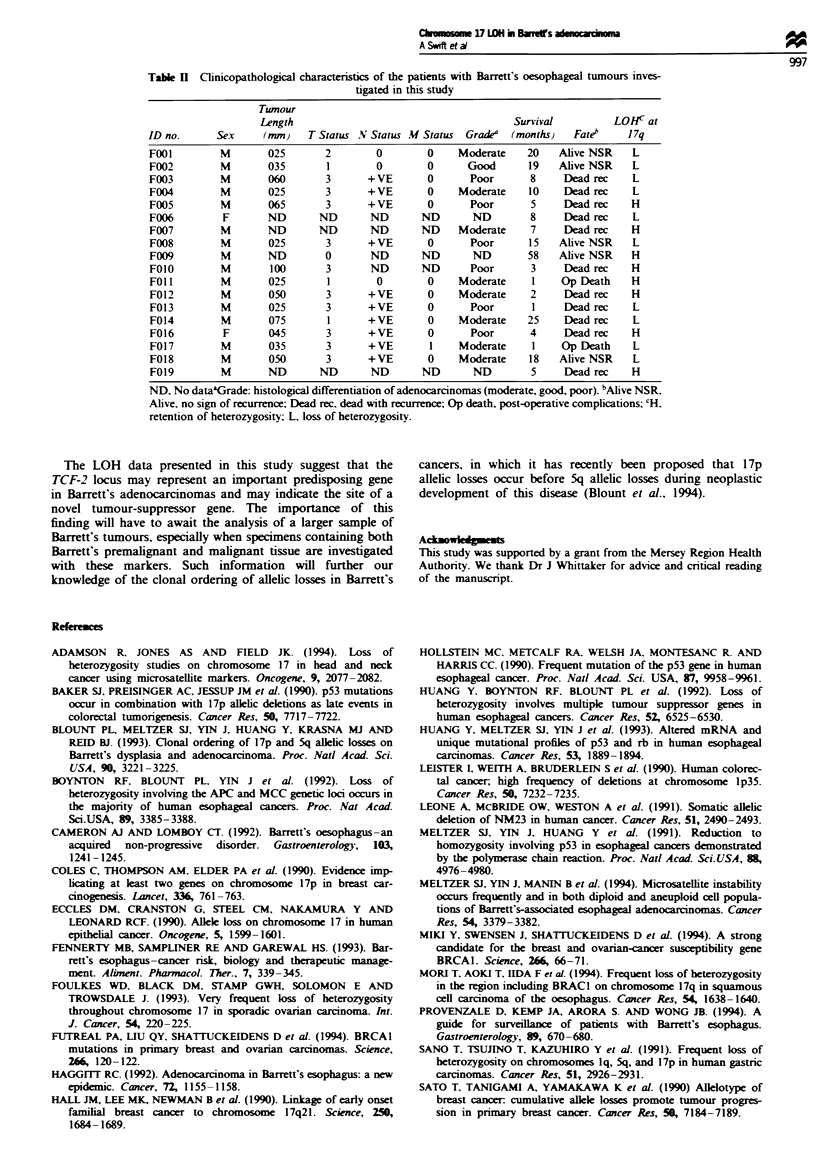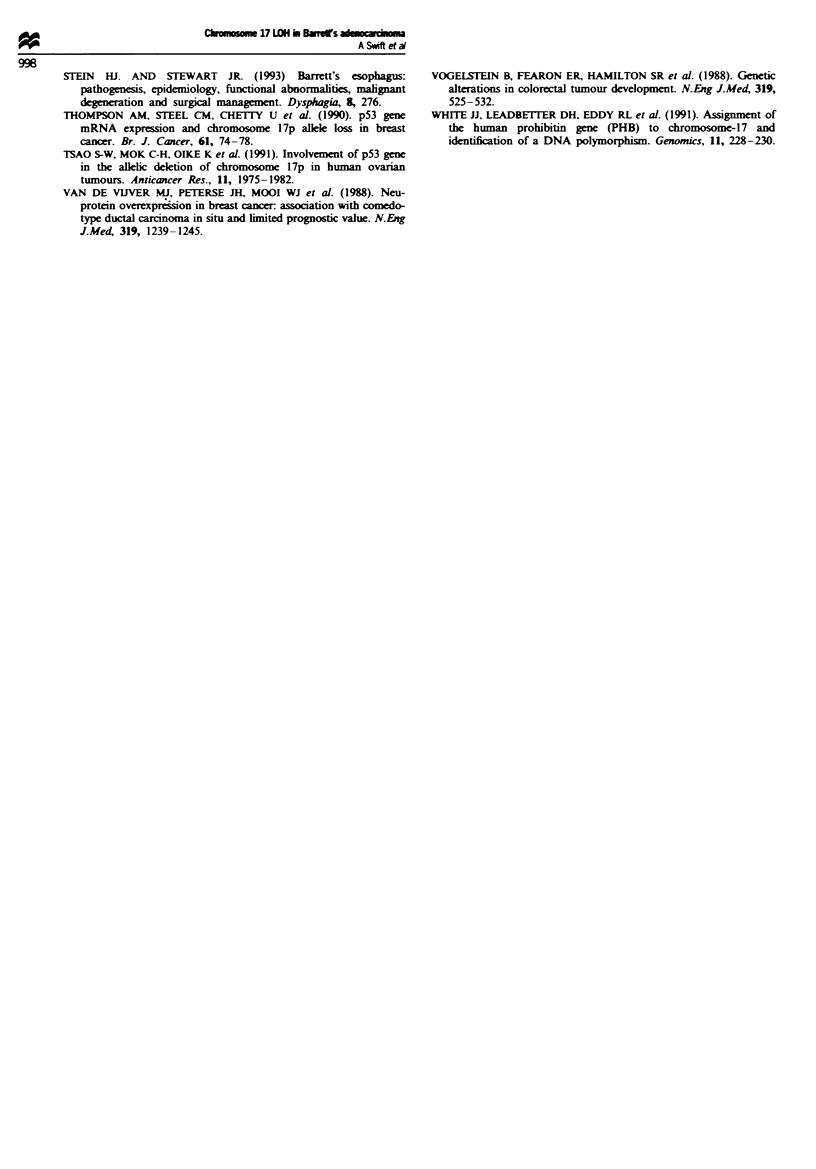# Frequent loss of heterozygosity on chromosome 17 at 17q11.2-q12 in Barrett's adenocarcinoma.

**DOI:** 10.1038/bjc.1995.191

**Published:** 1995-05

**Authors:** A. Swift, J. M. Risk, A. N. Kingsnorth, T. A. Wright, M. Myskow, J. K. Field

**Affiliations:** Department of Clinical Dental Sciences, University of Liverpool, UK.

## Abstract

Allelic loss on chromosome 17 in 18 Barrett's oesophageal tumours was analysed with 17 polymorphic microsatellite markers. Loss of heterozygosity (LOH) of one or more markers was seen in 72% (13 of 18) tumours on 17p and 56% (10 of 18) on 17q. The highest 17p losses were found at D17S799 (62%, five of eight) and D17S261 (55%, five of nine), while loss at the p53 locus was 31% (5 of 16). The highest loss on 17q was found at the TCF-2 (17q11.2-q12) locus with 66% (8 of 12) LOH. TCF-2 was the only marker lost in two of the tumour samples; furthermore, TCF-2 was lost in four other tumours which retained heterozygosity at the markers on either side of it, D17S261 and D17S740. Six markers were used to assess LOH at 17q11.2-q12, and five of eight of the tumour specimens which had LOH at TCF-2 had no other loss on 17q. No statistically significant correlations were found between loss on 17q or 17p and any clinicopathological parameters. We propose from these data that the 17q11.2-q12 region contains a novel predisposing gene in Barrett's adenocarcinomas and may represent the site of a tumour-suppressor gene.


© 1995 Stockton Press All rights reserved 0007-0920/95 $12.00

SHORT COMMUNICATION

### Frequent loss of heterozygosity on chromosome 17 at 17q11.2-q12 in Barrett's adenocarcinoma

A Swift[1], JM Risk[1], AN Kingsnorth[2], TA Wright[2], M Myskow[3] and JK Field[1]

[1]*Molecular Genetics and Oncology Group, Department of Clinical Dental Sciences and* [2]*Department of Surgery, The University of Liverpool, PO Box 147, Liverpool L69 3BX, UK;* [3]*Department of Pathology, Broadgreen Hospital, Liverpool, UK.*

**Summary** Allelic loss on chromosome 17 in 18 Barrett's oesophageal tumours was analysed with 17 polymorphic microsatellite markers. Loss of heterozygosity (LOH) of one or more markers was seen in 72% (13 of 18) tumours on 17p and 56% (10 of 18) on 17q. The highest 17p losses were found at *D17S799* (62%, five of eight) and *D17S261* (55%, five of nine), while loss at the p53 locus was 31% (5 of 16). The highest loss on 17q was found at the *TCF-2* (17q11.2-q12) locus with 66% (8 of 12) LOH. *TCF-2* was the only marker lost in two of the tumour samples; furthermore, *TCF-2* was lost in four other tumours which retained heterozygosity at the markers on either side of it, *D17S261* and *D17S740*. Six markers were used to assess LOH at 17q11.2-q12, and five of eight of the tumour specimens which had LOH at *TCF-2* had no other loss on 17q. No statistically significant correlations were found between loss on 17q or 17p and any clinicopathological parameters. We propose from these data that the 17q11.2-q12 region contains a novel predisposing gene in Barrett's adenocarcinomas and may represent the site of a tumour-suppressor gene.

**Keywords:** Barrett's adenocarcinoma; chromosome 17; loss of heterozygosity

Barrett's columnar metaplasia of the squamous epithelium of the oesophagus is a consequence of chronic gastro-oesophageal reflux. It has been estimated that approximately 700 000 people in the United States have acquired Barrett's oesophagus (Provenzale *et al.*, 1994). The risk of developing adenocarcinoma of the oesophagus in these patients is 30- to 40-fold higher than in the general population (Fennerty *et al.*, 1993; Stein and Stewart *et al.*, 1993). Once diagnosed, many patients with Barrett's oesophagus are entered into surveillance programmes in order to detect histopathological evidence of premalignant states, such as low-grade and high-grade dysplasia. Oesophagectomy for those observed to have early invasive carcinoma or high-grade dysplasia during such surveillance programmes results in improved survival.

A clear sequence from low-grade dysplasia to high-grade dysplasia to invasive carcinoma is observed to develop over a substantial period of perhaps 3-5 years (Cameron and Lomboy, 1992). During the last two decades the incidence of adenocarcinoma of the oesophagus has increased at a rate exceeding that of any other cancer, with an incidence of 500 cancers per 100 000 patients with Barrett's metaplasia per year (Haggitt, 1992).

Conventional histopathology with the detection of dysplasia is currently the only means of early diagnosis of Barrett's cancers. Oesophageal cancers share a number of molecular markers previously found in colorectal and gastric cancers, especially loss of heterozygosity (LOH) in chromosomes 5 and 17 (Vogelstein *et al.*, 1988; Leister *et al.*, 1990; Meltzer *et al.*, 1991; Sano *et al.*, 1991; Boynton *et al.*, 1992; Huang *et al.*, 1992; Blount *et al.*, 1993; Meltzer *et al.*, 1994). In addition, overexpression and mutations of the p53 tumour-suppressor gene are a frequent event in these tumours (Baker *et al.*, 1990; Hollstein *et al.*, 1990; Huang *et al.*, 1993), and microsatellite instability has recently been demonstrated in Barrett's cancers by Meltzer *et al.*, (1994). To date, the majority of investigations into LOH on chromosome 17 in Barrett's cancers have concentrated on the region containing the p53 gene. We have undertaken a detailed analysis of both chromosome 17 arms, using 17 microsatellite markers. The results of this investigation indicate the highest loss of heterozygosity on the q arm of chromosome 17 at 17q11.2-q12.

Correspondence: JK Field
Received 2 November 1994; revised 19 December 1994; accepted 21 December 1994

### Materials and Methods

### *Specimens*

Eighteen Barrett's oesophageal tumour specimens were collected at the Royal Liverpool University Hospital, Department of Surgery, and at the Cardiothoracic Centre, Liverpool. Tumour samples obtained from surgical specimens were frozen in liquid nitrogen and stored at $-70°C$. The pathology of all these specimens was assessed by MM. The sections were dissected to yield more than 50% tumour cells for polymerase chain reaction (PCR) analysis.

### *DNA extraction*

Genomic DNA was extracted from tumour specimens using the Nucleon II DNA extraction kit (Scotlab) following the manufacturer's instructions. Genomic DNA samples were stored at 4°C.

### *PCR and LOH analysis*

Microsatellite repeat primers were obtained from Isogen (The Netherlands). PCR reactions were performed in a 25 $\mu l$ reaction volume and contained 200 ng of genomic DNA, 200 $\mu M$ dNTP, 5 pmol each of forward and reverse primers, 0.5 units of *Taq* polymerase (Advanced Biotechnologies) and 2.5 $\mu l$ of $10\times$ buffer [670 mM Tris-HCl pH 8.5, 166 mM ammonium sulphate; 67 mM magnesium chloride; 1.7 mg $ml^{-1}$ bovine serum albumin (BSA); 100 $\mu M$ $\beta$-mercaptoethanol; 1% (w/v) Triton X-100]. The reactions were denatured for 5 min at 95°C and then the DNA was amplified for 30 cycles of 95°C for 30 s, 57°C for 30 s and 72°C for 30 s. A 10 $\mu l$ volume of the PCR product was electrophoresed for 10 h on a 10% polyacrylamide gel at 250 V and viewed by silver staining.

### Results

We have undertaken a LOH study on 18 Barrett's oesophageal tumours on chromosome 17, using 17 polymorphic microsatellite markers (Table I), in order to ascertain common regions of deletions on both arms of this chromosome and evaluate whether allelic loss was concentrated in any particular region. Loss of one or more markers was seen in 72% (13 of 18) of the specimens on 17p and in 56% (10 of 18) on 17q.

Significantly high frequencies of LOH (ie. > 30%) on 17p were found at *D17S799* (63%, five of eight) and *D17S261* (55%, five of nine), while loss of the p53 marker, *TP53*, usually occurred with one or all of the three markers located centromeric to it (*D17S520, D17S799, CHRNB1*), and was the sole 17p locus lost in only one case (patient 3). The highest loss on 17q was found to be at the *TCF-2* locus, with 66% LOH (8 of 12). It is of note that the *TCF-2* marker was the only marker lost in two of the Barrett's oesophageal tumours (patients 8 and 18). Furthermore, *TCF-2* was lost in four tumours which retained heterozygosity at informative markers on either side of it.

We have used six markers in the 17q11.2–q12 region as assigned by linkage mapping and show that five of eight of those Barrett's oesophageal tumours which have a loss at *TCF-2* have no other losses on 17q. Figure 1 demonstrates diagrammatically the region of minimal loss at the *TCF-2* locus at 17q11.2–q12. No correlation was found between loss on 17p or 17q and any clinicopathological parameters or survival (Table II). Also, no clinical correlations were found between loss at the *TCF-2* locus and any clinical parameters or survival.

**Table I** Loss of heterozygosity on chromosome 17 in Barrett's oesophageal tumours

| *Map localisation* | *Marker* | *Loss/informative/ no. analysed* | *Loss(%)* |
|---|---|---|---|
| 17p13.3–q11 | D17S578 | 2/8/16 | 25 |
| 17p13.1 | TP53 | 5/16/16 | 31 |
| 17p13–p12 | D17S520 | 6/13/18 | 46 |
| 17p13.1–p12 | D17S799 | 5/8/14 | 62 |
| 17p12–p11.1 | CHRNB1 | 6/13/18 | 46 |
| 17p12–p11.2 | D17S122 | 3/12/18 | 25 |
| 17p12–p11.2 | D17S261 | 5/9/14 | 55 |
| 17q11.2–q12 | TCF2 | 8/12/18 | 66 |
| 17q | D17S740 | 2/6/13 | 33 |
| 17q | D17S783 | 0/9/14 | 0 |
| 17q | D17S798 | 0/12/14 | 0 |
| 17q11.2–q12 | D17S250 | 2/11/11 | 18 |
| 17q11.2–q12 | THRA1 | 2/8/11 | 25 |
| 17q21.32 | GP3A | 2/8/18 | 25 |
| 17q21.3–q23 | MPO | 1/9/18 | 11 |
| 17q | D17S940 | 0/4/13 | 0 |
| 17q23–q25 | D17S515 | 0/13/18 | 0 |

### Discussion

We have detected a high incidence of loss of heterozygosity at the *TCF-2* locus (17q11.2–q12) on the q arm of chromosome 17. Loss at this site has not been previously reported in any oesophageal tumours including Barrett's adenocarcinoma. Blount *et al.* (1993) reported 17p deletions in 12/13 (92%) Barrett's oesophageal tumour specimens, while we have found 72% (13/18) LOH on the 17p arm. In comparison, on 17q we now report 56% (10/18) LOH, whereas there are no previous reports of LOH on 17q in Barrett's adenocarcinoma. In none of these cases was the entire 17q arm lost, whereas 56% had partial or interstitial deletions on 17q. The nearest similar study is that of Mori *et al.* (1994), who studied losses on 17q in squamous cell carcinomas of the oesophagus. Their investigation centred around the *BRCA1* region located telomeric to that in which we are interested and the marker nearest to the *TCF-2* locus they used was C117-316 (17q12–q21.1), which had a low LOH frequency.

There have been a number of investigations of other tumour types suggesting that there may be novel tumour-suppressor genes on both 17p and 17q. Apart from the p53 gene, several groups have reported the presence of a further gene at 17p13.3 in breast cancer (Coles *et al.*, 1990; Sato *et al.*, 1990; Thompson *et al.*, 1990), a finding also seen in ovarian tumours (Eccles *et al.*, 1990; Tsao *et al.*, 1991; Foulkes *et al.*, 1993). We have recently described the site of another putative tumour-suppressor gene in head and neck squamous cell carcinomas at *CHRNB1* (17p12–p11.1) (Adamson *et al.*, 1994). Furthermore, a number of genes on 17q have previously been implicated in breast cancer, including *BRCA1*, *NM23* and prohibitin (Hall *et al.*, 1990; Leone *et al.*, 1991; White *et al.*, 1991; Futreal *et al.*, 1994; Miki *et al.*, 1994). To this can be added the oncogene c-*erb*B-2 (17q12), which most likely acts by increasing copy number (Van de Vijver *et al.*, 1988). There are a number of possible candidate genes which have been assigned to the 17q11.2–q12 region, and these include *NF1* (neurofibromin 1), *CSF3* (colony-stimulating factor 3), *erb*B-2 (epidermal growth factor) and *ITB4* (integrin $\beta_4$).

**Figure 1** Schematic diagram of the microsatellite markers analysed on chromosome 17 and the patterns of losses for eight cases of Barrett's adenocarcinoma. The markers are listed in order according to the sex-averaged genetic map from the GDB (UK) database. Minimal area of loss on 17q is shown.

**Table II** Clinicopathological characteristics of the patients with Barrett's oesophageal tumours investigated in this study

| ID no. | Sex | Tumour Length (mm) | T Status | N Status | M Status | Grade[a] | Survival (months) | Fate[b] | LOH[c] at 17q |
|---|---|---|---|---|---|---|---|---|---|
| F001 | M | 025 | 2 | 0 | 0 | Moderate | 20 | Alive NSR | L |
| F002 | M | 035 | 1 | 0 | 0 | Good | 19 | Alive NSR | L |
| F003 | M | 060 | 3 | +VE | 0 | Poor | 8 | Dead rec | L |
| F004 | M | 025 | 3 | +VE | 0 | Moderate | 10 | Dead rec | L |
| F005 | M | 065 | 3 | +VE | 0 | Poor | 5 | Dead rec | H |
| F006 | F | ND | ND | ND | ND | ND | 8 | Dead rec | L |
| F007 | M | ND | ND | ND | ND | Moderate | 7 | Dead rec | H |
| F008 | M | 025 | 3 | +VE | 0 | Poor | 15 | Alive NSR | L |
| F009 | M | ND | 0 | ND | ND | ND | 58 | Alive NSR | H |
| F010 | M | 100 | 3 | ND | ND | Poor | 3 | Dead rec | H |
| F011 | M | 025 | 1 | 0 | 0 | Moderate | 1 | Op Death | H |
| F012 | M | 050 | 3 | +VE | 0 | Moderate | 2 | Dead rec | H |
| F013 | M | 025 | 3 | +VE | 0 | Poor | 1 | Dead rec | L |
| F014 | M | 075 | 1 | +VE | 0 | Moderate | 25 | Dead rec | L |
| F016 | F | 045 | 3 | +VE | 0 | Poor | 4 | Dead rec | H |
| F017 | M | 035 | 3 | +VE | 1 | Moderate | 1 | Op Death | L |
| F018 | M | 050 | 3 | +VE | 0 | Moderate | 18 | Alive NSR | L |
| F019 | M | ND | ND | ND | ND | ND | 5 | Dead rec | H |

ND, No data[a]Grade: histological differentiation of adenocarcinomas (moderate, good, poor). [b]Alive NSR, Alive, no sign of recurrence; Dead rec, dead with recurrence; Op death, post-operative complications; [c]H, retention of heterozygosity; L, loss of heterozygosity.

The LOH data presented in this study suggest that the *TCF-2* locus may represent an important predisposing gene in Barrett's adenocarcinomas and may indicate the site of a novel tumour-suppressor gene. The importance of this finding will have to await the analysis of a larger sample of Barrett's tumours, especially when specimens containing both Barrett's premalignant and malignant tissue are investigated with these markers. Such information will further our knowledge of the clonal ordering of allelic losses in Barrett's cancers, in which it has recently been proposed that 17p allelic losses occur before 5q allelic losses during neoplastic development of this disease (Blount *et al.*, 1994).

**Acknowledgements**
This study was supported by a grant from the Mersey Region Health Authority. We thank Dr J Whittaker for advice and critical reading of the manuscript.

**References**

ADAMSON R, JONES AS AND FIELD JK. (1994). Loss of heterozygosity studies on chromosome 17 in head and neck cancer using microsatellite markers. *Oncogene*, **9**, 2077–2082.
BAKER SJ, PREISINGER AC, JESSUP JM *et al.* (1990). p53 mutations occur in combination with 17p allelic deletions as late events in colorectal tumorigenesis. *Cancer Res*, **50**, 7717–7722.
BLOUNT PL, MELTZER SJ, YIN J, HUANG Y, KRASNA MJ AND REID BJ. (1993). Clonal ordering of 17p and 5q allelic losses on Barrett's dysplasia and adenocarcinoma. *Proc. Natl Acad. Sci. USA*, **90**, 3221–3225.
BOYNTON RF, BLOUNT PL, YIN J *et al.* (1992). Loss of heterozygosity involving the APC and MCC genetic loci occurs in the majority of human esophageal cancers. *Proc. Nat Acad. Sci.USA*, **89**, 3385–3388.
CAMERON AJ AND LOMBOY CT. (1992). Barrett's oesophagus–an acquired non-progressive disorder. *Gastroenterology*, **103**, 1241–1245.
COLES C, THOMPSON AM, ELDER PA *et al.* (1990). Evidence implicating at least two genes on chromosome 17p in breast carcinogenesis. *Lancet*, **336**, 761–763.
ECCLES DM, CRANSTON G, STEEL CM, NAKAMURA Y AND LEONARD RCF. (1990). Allele loss on chromosome 17 in human epithelial cancer. *Oncogene*, **5**, 1599–1601.
FENNERTY MB, SAMPLINER RE AND GAREWAL HS. (1993). Barrett's esophagus–cancer risk, biology and therapeutic management. *Aliment. Pharmacol. Ther.*, **7**, 339–345.
FOULKES WD, BLACK DM, STAMP GWH, SOLOMON E AND TROWSDALE J. (1993). Very frequent loss of heterozygosity throughout chromosome 17 in sporadic ovarian carcinoma. *Int. J. Cancer*, **54**, 220–225.
FUTREAL PA, LIU QY, SHATTUCKEIDENS D *et al.* (1994). BRCA1 mutations in primary breast and ovarian carcinomas. *Science*, **266**, 120–122.
HAGGITT RC. (1992). Adenocarcinoma in Barrett's esophagus: a new epidemic. *Cancer*, **72**, 1155–1158.
HALL JM, LEE MK, NEWMAN B *et al.* (1990). Linkage of early onset familial breast cancer to chromosome 17q21. *Science*, **250**, 1684–1689.
HOLLSTEIN MC, METCALF RA, WELSH JA, MONTESANC R. AND HARRIS CC. (1990). Frequent mutation of the p53 gene in human esophageal cancer. *Proc. Natl Acad. Sci.* USA, **87**, 9958–9961.
HUANG Y, BOYNTON RF, BLOUNT PL *et al.* (1992). Loss of heterozygosity involves multiple tumour suppressor genes in human esophageal cancers. *Cancer Res*, **52**, 6525–6530.
HUANG Y, MELTZER SJ, YIN J *et al.* (1993). Altered mRNA and unique mutational profiles of p53 and rb in human esophageal carcinomas. *Cancer Res*, **53**, 1889–1894.
LEISTER I, WEITH A, BRUDERLEIN S *et al.* (1990). Human colorectal cancer; high frequency of deletions at chromosome 1p35. *Cancer Res*, **50**, 7232–7235.
LEONE A, MCBRIDE OW, WESTON A *et al.* (1991). Somatic allelic deletion of NM23 in human cancer. *Cancer Res*, **51**, 2490–2493.
MELTZER SJ, YIN J, HUANG Y *et al.* (1991). Reduction to homozygosity involving p53 in esophageal cancers demonstrated by the polymerase chain reaction. *Proc. Natl Acad. Sci.USA*, **88**, 4976–4980.
MELTZER SJ, YIN J, MANIN B *et al.* (1994). Microsatellite instability occurs frequently and in both diploid and aneuploid cell populations of Barrett's-associated esophageal adenocarcinomas. *Cancer Res*, **54**, 3379–3382.
MIKI Y, SWENSEN J, SHATTUCKEIDENS D *et al.* (1994). A strong candidate for the breast and ovarian-cancer susceptibility gene BRCA1. *Science*, **266**, 66–71.
MORI T, AOKI T, IIDA F *et al.* (1994). Frequent loss of heterozygosity in the region including BRAC1 on chromosome 17q in squamous cell carcinoma of the oesophagus. *Cancer Res*, **54**, 1638–1640.
PROVENZALE D, KEMP JA, ARORA S. AND WONG JB. (1994). A guide for surveillance of patients with Barrett's esophagus. *Gastroenterology*, **89**, 670–680.
SANO T, TSUJINO T, KAZUHIRO Y *et al.* (1991). Frequent loss of heterozygosity on chromosomes 1q, 5q, and 17p in human gastric carcinomas. *Cancer Res*, **51**, 2926–2931.
SATO T, TANIGAMI A, YAMAKAWA K *et al.* (1990) Allelotype of breast cancer: cumulative allele losses promote tumour progression in primary breast cancer. *Cancer Res*, **50**, 7184–7189.

STEIN HJ. AND STEWART JR. (1993) Barrett's esophagus: pathogenesis, epidemiology, functional abnormalities, malignant degeneration and surgical management. *Dysphagia*, **8**, 276.

THOMPSON AM, STEEL CM, CHETTY U *et al.* (1990). p53 gene mRNA expression and chromosome 17p allele loss in breast cancer. *Br. J. Cancer*, **61**, 74–78.

TSAO S-W, MOK C-H, OIKE K *et al.* (1991). Involvement of p53 gene in the allelic deletion of chromosome 17p in human ovarian tumours. *Anticancer Res.*, **11**, 1975–1982.

VAN DE VIJVER MJ, PETERSE JH, MOOI WJ *et al.* (1988). Neu-protein overexpression in breast cancer: association with comedo-type ductal carcinoma in situ and limited prognostic value. *N.Eng J.Med*, **319**, 1239–1245.

VOGELSTEIN B, FEARON ER, HAMILTON SR *et al.* (1988). Genetic alterations in colorectal tumour development. *N.Eng J.Med*, **319**, 525–532.

WHITE JJ, LEADBETTER DH, EDDY RL *et al.* (1991). Assignment of the human prohibitin gene (PHB) to chromosome-17 and identification of a DNA polymorphism. *Genomics*, **11**, 228–230.